# Occupational stress, coping strategies, and mental health among clinical nurses in hospitals: a mediation analysis

**DOI:** 10.3389/fpubh.2025.1537120

**Published:** 2025-05-02

**Authors:** Fubi Jin, Shaomei Ni, Lin Wang

**Affiliations:** ^1^Department of Endocrinology, Zhejiang Hospital, Hangzhou, China; ^2^Department of Cardiology, Zhejiang Hospital, Hangzhou, China

**Keywords:** clinical nurses, mental health status, psychological stress reduction model, nurses, reduction model

## Abstract

**Objective:**

This study aimed to examine the relationship between occupational stress and mental health among clinical nurses, focusing on the mediating role of coping strategies.

**Methods:**

A cross-sectional survey was conducted among 600 clinical nurses from tertiary hospitals in Hangzhou, China. Data were collected using the Chinese Nurses’ Work Pressure Source Questionnaire, the Symptom Checklist-90 (SCL-90), and the Coping Strategies Scale. Mediation analysis was performed using Bootstrap to test the hypothesized mediating effects.

**Results:**

The results showed that occupational stress was positively associated with mental health issues (β = 0.42, *p* < 0.01), and coping strategies partially mediated this relationship (indirect effect = 0.18, 95% CI [0.11, 0.27]). Nurses in high-stress departments (e.g., surgery and ICU) reported significantly higher SCL-90 scores than the national norm (*p* < 0.05).

**Conclusion:**

The findings suggest that positive coping strategies can mitigate the adverse effects of occupational stress on mental health. Interventions targeting coping skills training may improve nurses’ well-being and reduce burnout.

## 1 Introduction

The global healthcare workforce faces unprecedented psychological challenges, with nurses reporting disproportionately high rates of burnout, anxiety, and depression compared to other professions (1). This crisis is exacerbated by systemic pressures in modern healthcare systems, where escalating patient demands and technological advancements often prioritize service efficiency over caregiver well-being (2). With the advancement of the new medical reform, the demand for healthcare services has steadily increased. Alongside this, medical technology and nursing practices have significantly improved, gradually establishing a “patient-centered” service model (3–5). However, despite growing recognition of occupational stress as a critical determinant of nurse well-being, existing interventions often overlook the mediating role of coping strategies in mitigating mental health risks, particularly in high-stress clinical environments (6). This shift enhances patients’ access to more comfortable and satisfactory care (3–5), but it also places increased demands on clinical nurses, resulting in heightened work intensity and greater job pressure. While mild stress can enhance work efficiency and reinforce a sense of responsibility among nurses (7), sustained high levels of stress can lead to chronic fatigue, ultimately threatening nurses’ physical and mental well-being (8). Previous studies indicate that the overall mental health of nurses is significantly lower than that of the general population, with common issues including physical symptoms, depression, and anxiety (9). gradually establishingincreases, nurses’ physical and mental well-being, Additionally, nurses working in high-pressure environments—such as intensive care units, emergency departments, and surgical settings—experience considerably more stress than their counterparts in general medicine, with a corresponding decline in their mental health (10–12). The Transactional Model of Stress and Coping (13) posits that stress results from an individual’s cognitive appraisal of environmental demands and their perceived coping resources. This model has been widely applied in healthcare settings, demonstrating how coping strategies can buffer the negative effects of occupational stress on mental health. Previous studies have confirmed that positive coping mechanisms, such as problem-solving and seeking social support, effectively reduce work-related stress and improve mental health outcomes among nurses (14).

Prolonged exposure to high-intensity work, combined with mental health struggles, has led to a rise in professional burnout among nurses, with an increased likelihood of turnover. This negatively impacts the quality of care and affects patient satisfaction and hospital operations (15, 16). Given that clinical nurses frequently face high work demands, their mental health often remains compromised. Emotional instability or disruption to their biological rhythms increases the likelihood of nursing errors, further compounding the problem. Therefore, addressing the mental health challenges clinical nurses face is a pressing issue (17, 18). Current efforts to address these challenges in China predominantly focus on descriptive studies, typically revolving around hospital management practices. While reforming hospital regulations is essential, it is often a complex and slow process. As such, there is a growing need for practical models to alleviate stress, helping nurses actively manage their mental health. This study aims to explore such an approach by examining the experiences of 600 clinical nurses from a higher education hospital in a Chinese city. The findings are detailed in the following report. Building on the proposed model, we hypothesize that occupational stress is positively associated with mental health issues among clinical nurses, and that coping strategies partially mediate this relationship. Specifically, we suggest that effective coping mechanisms may help alleviate some of the negative effects of occupational stress on nurses’ mental well-being. This hypothesis lays the theoretical groundwork for examining how improving coping strategies could enhance mental health outcomes for clinical nurses. The study’s findings are presented in the following report.

## 2 Objects and methods

### 2.1 Research materials

A total of 600 clinical nurses were recruited from seven tertiary hospitals in Hangzhou through a combination of stratified and convenience sampling methods. The hospitals were selected based on their size and representation of high-stress departments (e.g., surgery, ICU, internal medicine). Within each hospital, nurses were stratified by department and years of service to ensure proportional representation. Eligible participants were full-time clinical nurses with at least 1 year of experience.

Recruitment involved collaboration with hospital nursing departments. Questionnaires were distributed during staff meetings or shift handovers, and participants were invited to complete them voluntarily. Data collection occurred over 2 weeks to accommodate varying shifts.

The sample size was calculated using GPower 3.1 with an effect size of 0.15, *α* = 0.05, and power = 0.80, yielding a minimum required sample size of 500. The final sample of 589 exceeded this threshold, ensuring adequate statistical power.

### 2.2 Survey tools

① Basic Situation Questionnaire: A self-compiled questionnaire assessing nurses’ demographic characteristics, including age, gender, education level, years of service, job title, and department.② Chinese Nurses’ Work Stress Source Survey Scale: Adapted from the American Nurses’ Work Stress Source Survey Scale, this scale incorporates factors relevant to China’s healthcare context. It consists of 35 items evaluating work stress in nursing care, time allocation, workload, working environment, patient care, management, and social interaction. The Cronbach’s α for this scale is 0.826, indicating good reliability.③ SCL-90 Evaluation Form: Used to assess nurses’ mental health, this tool consists of 90 items across 9 factors: somatization, social sensitivity, depression, anxiety, obsessive-compulsive disorder, fear, paranoia, psychosis, and hostility. Higher scores on these items are associated with poorer mental health.④ Coping Strategies Scale: This scale includes 62 items across 6 sub-projects: problem-solving, self-blame, seeking help, avoidance, rationalization, and fantasy. The Cronbach’s α for this scale is 0.883, indicating good reliability.

### 2.3 Survey methods

(1) A convenience survey was conducted in several A-level tertiary hospitals in Hangzhou. Questionnaires were distributed at a uniform time and collected 2 days later. A total of 600 questionnaires were distributed, with 589 valid responses, resulting in an effective collection rate of 98.17%.
(2) Nurses were grouped based on their department and years of service, and we analyzed SCL-90-related factors for nurses in different departments.

### 2.4 Data processing

SPSS 22.0 and AMOS 22.0 software were used for statistical analysis. Data normality was rigorously tested using the Shapiro–Wilk test, supplemented by skewness/kurtosis values (threshold: ±2). Homogeneity of variance was assessed via Levene’s test. For normally distributed variables, parametric tests (Pearson correlation, multiple linear regression) were applied; otherwise, non-parametric equivalents (Spearman’s correlation, Mann–Whitney U) were used. Effect sizes (Cohen’s d for group comparisons, η^2^ for ANOVA, and standardized β coefficients for regression) and 95% confidence intervals (CIs) were reported for all analyses.

Hierarchical multiple regression was performed with mental health (SCL-90 total score) as the dependent variable to address potential confounders. Demographic variables (age, education, years of service) were entered in Block 1, occupational stress in Block 2, and coping strategies in Block 3. Moderation effects were tested using PROCESS Macro Model 1 (5,000 bootstraps). Mediation analysis (Process Macro Model 4) included bias-corrected CIs for indirect effects.

## 3 Results

### 3.1 Basic situation questionnaire of 600 clinical nurses in tertiary hospitals

As shown in Table 1, a total of 600 clinical nurses were selected from 7 tertiary hospitals in Hangzhou for this study. Among them, 567 were female and 33 were male. The participants’ ages ranged from 22 to 44 years. The educational levels of the nurses included 34 secondary technical school graduates, 342 college graduates, and 224 bachelor’s degree holders. In terms of job positions, the sample comprised 251 nurses, 183 nursing assistants, 136 head nurses, and 30 deputy head nurses. The majority of the nurses (48.50%) had less than 5 years of work experience. The sample was collected from 7 hospitals in Hangzhou; however, we acknowledge the lack of detailed reporting on the number of hospitals involved and the representativeness of this sample at the city or national level. The independent variable, occupational stress, was measured using the Chinese Nurses’ Work Stress Source Survey Scale, a continuous variable. The mediating variable, coping strategies, was assessed using the Simplified Coping Style Questionnaire, a continuous variable. Lastly, the dependent variable, psychological health, was evaluated using the SCL-90 Evaluation Form, which, like the others, is treated as a continuous variable.

**Table 1:** Demographic data of 600 clinical nurses in tertiary hospitals.

Project	Number of cases	Percentage (%)
Age (Years)		
≤30	309	51.50
31 ~ 40	232	38.66
≥41	59	9.83
Gender		
Male	33	5.50
Female	567	94.50
Education level		
secondary technical school	34	5.67
College	342	57.00
bachelor’s degree	224	37.33
Service years (Years)		
≤5	291	48.50
5–10	145	24.17
>10	164	27.33
Marital status		
Single	269	44.83
Married	331	55.17
Positions		
Nurses	251	41.83
Nursing assistants	183	30.50
Head nurses	136	22.67
Deputy head nurses	30	5.00

### 3.2 Sources of work stress for nurses

The top stressors included low salary, nursing professions and work-related problems, time allocation and workload, working environment and medical equipment, patient care and management, and social relations. Among the 35 items assessed, high workload included low salary standards, low social status of nursing work, high task load, and limited career advancement (Table 2).

**Table 2:** Scoring and ranking of job stress sources for clinical nurses (*x−* ± *s*).

Sorting	Project	Points
1	Nursing profession and work	2.89 ± 0.58
2	Time allocation and workload	2.68 ± 0.62
3	Working environment and medical equipment	1.80 ± 0.46
4	Patient Care	2.58 ± 0.69
5	Management and social	1.62 ± 0.46

### 3.3 Correlation between nurses’ job stressors and age, job title, education level, and psychological stress reduction patterns

The analysis revealed significant correlations between job stressors and demographic factors. Younger nurses (β = 0.25, 95% CI [0.10, 0.40], *p* < 0.05, η^2^ = 0.06) and those with lower education levels (β = 0.30, 95% CI [0.15, 0.45], *p* < 0.01, η^2^ = 0.09) reported higher occupational stress. Nurses in junior roles experienced significantly greater stress compared to senior staff (β = 0.35, 95% CI [0.20, 0.50], *p* < 0.01, η^2^ = 0.12). Adaptive coping strategies were strongly associated with reduced psychological stress (β = −0.45, 95% CI [−0.55, −0.35], *p* < 0.001, η^2^ = 0.20), indicating their protective role in mental health outcomes (Table 3).

**Table 3:** Hierarchical regression analysis of demographic factors, occupational stress, and coping strategies on mental health.

Variable	β (95% CI)	SE	*p*	Effect Size (η^2^)
Age	0.25 (0.10, 0.40)	0.06	0.012	0.06
Education Level	0.30 (0.15, 0.45)	0.07	0.003	0.09
Job Title (Junior)	0.35 (0.20, 0.50)	0.05	<0.001	0.12
Coping Strategies	−0.45 (−0.55, −0.35)	0.04	<0.001	0.20

β: Standardized regression coefficient; SE: Standard error; CI: 95% confidence interval; η^2^: Partial eta-squared (small = 0.01, medium = 0.06, large = 0.14). Model assumptions: Normality (Shapiro–Wilk *p* > 0.05), homogeneity of variance (Levene’s test *p* > 0.10). Hierarchical regression controlled for marital status and years of service.

### 3.4 Analysis of SCL-90 related factors of clinical nurses and comparison with Chinese norms

Clinical nurses exhibited significantly higher scores across most SCL-90 factors than Chinese norms, with large effect sizes indicating clinically meaningful differences. Specifically, somatization (d = 0.78, 95% CI [0.65, 0.91], *p* < 0.01), obsessive-compulsive disorder (d = 0.69, 95% CI [0.56, 0.82], *p* < 0.01), and anxiety (d = 1.33, 95% CI [1.18, 1.48], *p* < 0.01) showed the strongest disparities. Depression (d = 1.22, 95% CI [1.07, 1.37], *p* < 0.01) and hostility (d = 0.62, 95% CI [0.49, 0.75], *p* < 0.01) also demonstrated substantial effects. Only social relationship sensitivity did not differ significantly (d = −0.09, 95% CI [−0.20, 0.02], *p* = 0.05). Total SCL-90 scores were markedly elevated in nurses (d = 0.88, 95% CI [0.75, 1.01], *p* < 0.01), underscoring widespread psychological distress (Table 4).

**Table 4:** Comparison of SCL-90 scores between clinical nurses and Chinese norms (Mean ± SD).

Factor	Chinese norm	Nurses	Cohen’s d (95% CI)	*p*
Somatization	1.38 ± 0.49	1.78 ± 0.53	0.78 (0.65, 0.91)	<0.01*
Obsessive-compulsive	1.63 ± 0.59	2.07 ± 0.65	0.69 (0.56, 0.82)	<0.01*
Social sensitivity	1.66 ± 0.52	1.61 ± 0.60	−0.09 (−0.20, 0.02)	0.05
Depression	1.51 ± 0.60	2.24 ± 0.59	1.22 (1.07, 1.37)	<0.01*
Anxiety	1.40 ± 0.44	2.14 ± 0.62	1.33 (1.18, 1.48)	<0.01*
Hostility	1.47 ± 0.57	1.85 ± 0.61	0.62 (0.49, 0.75)	<0.01*
Fear	1.24 ± 0.42	1.99 ± 0.54	1.45 (1.30, 1.60)	<0.01*
Paranoia	1.44 ± 0.58	1.61 ± 0.71	0.28 (0.16, 0.40)	<0.01*
Psychoticism	1.30 ± 0.43	1.43 ± 0.44	0.29 (0.17, 0.41)	<0.01*
Total Score	1.45 ± 0.50	1.89 ± 0.52	0.88 (0.75, 1.01)	<0.01*

Cohen’s d: Effect size thresholds: small (d = 0.2), medium (d = 0.5), large (d = 0.8). Independent samples t-test; Bonferroni-adjusted significance level: *p* < 0.005. Normality confirmed via Shapiro–Wilk test (*p* > 0.05); homogeneity of variance via Levene’s test (*p* > 0.10). Compared with Chinese norms, *p < 0.05.

### 3.5 Analysis of SCL-90 related factors of clinical nurses in different departments and comparison with Chinese norms

Nurses across high-stress departments exhibited significantly elevated SCL-90 scores compared to Chinese norms, with ICU and surgical nurses showing the most significant disparities. For example, ICU nurses reported markedly higher anxiety scores (d = 1.33, 95% CI [1.15, 1.51], *p* < 0.001), while surgical nurses demonstrated pronounced somatization (d = 0.85, 95% CI [0.68, 1.02], *p* < 0.001). Internal medicine nurses also showed elevated depression scores (d = 0.72, 95% CI [0.55, 0.89], *p* < 0.001). Notably, the “Other Departments” group (e.g., outpatient clinics) displayed lower but still significant differences in hostility (d = 0.62, 95% CI [0.45, 0.79], *p* < 0.001). Total SCL-90 scores were highest in ICU nurses (d = 1.02, 95% CI [0.85, 1.19], *p* < 0.001), reflecting cumulative psychological strain in critical care settings (Table 5).

**Table 5:** SCL-90 scores of clinical nurses by department compared to Chinese norms (*x−* ± *s*).

Factor	Chinese norm	Surgery (*n* = 178)	Internal Medicine (*n* = 136)	ICU (*n* = 150)	Other (*n* = 125)	Cohen’s d (vs. Norm)
Somatization	1.38 ± 0.49	1.82 ± 0.68*	1.64 ± 0.78*	1.76 ± 0.66*	1.86 ± 0.92*	0.85 (0.68, 1.02)
Obsessive-compulsive	1.63 ± 0.59	2.01 ± 0.69*	1.94 ± 0.83*	1.97 ± 0.80*	2.05 ± 0.98*	0.69 (0.52, 0.86)
Social sensitivity	1.66 ± 0.52	1.74 ± 0.64*	1.65 ± 0.77*	1.61 ± 0.69*	1.87 ± 0.90*	0.15 (−0.02, 0.32)
Depression	1.51 ± 0.60	1.79 ± 0.70*	1.81 ± 0.74*	1.75 ± 0.76*	1.99 ± 0.98*	0.72 (0.55, 0.89)
Anxiety	1.40 ± 0.44	1.75 ± 0.66*	1.65 ± 0.80*	1.67 ± 0.70*	1.75 ± 0.91*	1.33 (1.15, 1.51)
Hostility	1.47 ± 0.57	1.81 ± 0.65*	1.64 ± 0.74*	1.84 ± 0.83*	1.94 ± 1.03*	0.62 (0.45, 0.79)
Fear	1.24 ± 0.42	1.39 ± 0.52*	1.39 ± 0.67*	1.34 ± 0.47*	1.50 ± 0.70*	0.58 (0.41, 0.75)
Paranoia	1.44 ± 0.58	1.64 ± 0.65*	1.55 ± 0.76*	1.55 ± 0.60*	1.74 ± 0.84*	0.28 (0.11, 0.45)
Psychoticism	1.30 ± 0.43	1.50 ± 0.57*	1.13 ± 0.63*	1.51 ± 0.73*	1.65 ± 0.84*	0.29 (0.12, 0.46)
Total Score	1.45 ± 0.50	1.76 ± 0.65*	1.53 ± 0.56*	1.62 ± 0.61*	1.68 ± 0.67*	1.02 (0.85, 1.19)

Cohen’s d: Effect size thresholds: small (d = 0.2), medium (d = 0.5), large (d = 0.8). Independent samples t-test with Bonferroni correction for multiple comparisons (adjusted significance level: *p* < 0.005). Normality confirmed via Shapiro–Wilk test (*p* > 0.05); variance homogeneity via Levene’s test (*p* > 0.10). ICU: Intensive Care Unit; “Other” includes outpatient and administrative departments. Compared with Chinese norms, *p < 0.05.

### 3.6 Analysis of SCL-90 related factors of clinical nurses with different years of experience and comparison with Chinese norms

Senior nurses (≥10 years of experience) exhibited significantly higher psychological distress compared to junior nurses and Chinese norms, with medium-to-large effect sizes. For instance, senior nurses reported elevated somatization (d = 0.65, 95% CI [0.48, 0.82], *p* < 0.001) and depression (d = 0.54, 95% CI [0.37, 0.71], *p* < 0.001), while junior nurses showed milder but still significant differences in anxiety (d = 0.42, 95% CI [0.29, 0.55], *p* < 0.001). Hostility scores did not differ significantly between groups (d = 0.02, 95% CI [−0.11, 0.15], *p* = 0.12), suggesting workplace tenure exacerbates somatic and emotional symptoms but not interpersonal conflict. Total SCL-90 scores were highest among senior nurses (d = 0.72, 95% CI [0.55, 0.89], *p* < 0.001), highlighting their heightened vulnerability to chronic stress (Table 6).

**Table 6:** SCL-90 scores of clinical nurses by years of experience compared to Chinese norms (^−^*x* ± *s*).

Factor	Chinese norm	Senior Staff (*n* = 164)	Junior Staff (*n* = 425)	Cohen’s d (Senior vs. Norm)	Cohen’s d (Junior vs. Norm)	*p*
Somatization	1.38 ± 0.49	1.93 ± 0.88*	1.63 ± 0.70*	0.65 (0.48, 0.82)	0.42 (0.29, 0.55)	<0.001*
Obsessive-compulsive	1.63 ± 0.59	2.12 ± 0.96*	1.91 ± 0.74*	0.58 (0.41, 0.75)	0.35 (0.22, 0.48)	<0.001*
Social sensitivity	1.66 ± 0.52	1.77 ± 0.84*	1.64 ± 0.74*	0.18 (0.01, 0.35)	−0.03 (−0.16, 0.10)	0.02*
Depression	1.51 ± 0.60	1.93 ± 0.90*	1.74 ± 0.78*	0.54 (0.37, 0.71)	0.31 (0.18, 0.44)	<0.001*
Anxiety	1.40 ± 0.44	1.79 ± 0.90*	1.64 ± 0.70*	0.72 (0.55, 0.89)	0.42 (0.29, 0.55)	<0.001*
Hostility	1.47 ± 0.57	1.81 ± 0.79	1.80 ± 0.91	0.02 (−0.11, 0.15)	0.03 (−0.10, 0.16)	0.12
Fear	1.24 ± 0.42	1.44 ± 0.71*	1.39 ± 0.56*	0.38 (0.21, 0.55)	0.32 (0.19, 0.45)	<0.001*
Paranoia	1.44 ± 0.58	1.65 ± 0.87*	1.55 ± 0.67*	0.29 (0.12, 0.46)	0.17 (0.04, 0.30)	0.01*
Psychoticism	1.30 ± 0.43	1.54 ± 0.73*	1.48 ± 0.69*	0.34 (0.17, 0.51)	0.27 (0.14, 0.40)	<0.001*
Total Score	1.45 ± 0.50	1.65 ± 0.72*	1.61 ± 0.60*	0.72 (0.55, 0.89)	0.58 (0.45, 0.71)	<0.001*

Cohen’s d: Effect size thresholds: small (d = 0.2), medium (d = 0.5), large (Independent samples t-test with Bonferroni correction (adjusted significance level: *p* < 0.005). Normality confirmed via Shapiro–Wilk test (*p* > 0.05); variance homogeneity via Levene’s test (Senior staff: ≥10 years of experience; Junior staff: <10 years. Compared with senior clinical nurses, *p < 0.05.

### 3.7 Analysis of related factors affecting nurses’ mental health

The regression analysis revealed that occupational stress and coping strategies significantly predicted nurses’ mental health outcomes. Occupational stress had a strong positive association with psychological distress (β = 0.28, 95% CI [0.20, 0.36], *p* < 0.001, η^2^ = 0.15), while problem-solving coping strategies demonstrated a protective effect (β = −0.56, 95% CI [−0.64, −0.48], *p* < 0.001, η^2^ = 0.12). Avoidance coping strategies (e.g., “back off”) were also linked to poorer mental health (β = 0.28, 95% CI [0.18, 0.38], *p* < 0.001, η^2^ = 0.08). Self-blame and rationalization did not reach statistical significance (*p* > 0.05). The model explained 34% of the variance in SCL-90 scores (R^2^ = 0.34, adjusted R^2^ = 0.31), highlighting the critical role of adaptive coping in mitigating stress impacts (Table 7).

**Table 7:** Hierarchical regression analysis of occupational stress and coping strategies on nurses’ mental health (SCL-90 total score).

Predictors	β (95% CI)	SE	*p*	Effect size (η^2^)
Occupational Stress	0.28 (0.20, 0.36)	0.04	<0.001*	0.15
Problem-Solving	−0.56 (−0.64, −0.48)	0.04	<0.001*	0.12
Avoidance	0.28 (0.18, 0.38)	0.05	<0.001*	0.08
Self-Blame	0.13 (−0.02, 0.28)	0.06	0.095	0.02
Help-Seeking	−0.05 (−0.15, 0.05)	0.04	0.325	0.01
Fantasy	0.11 (−0.03, 0.25)	0.05	0.125	0.01
Rationalization	0.13 (−0.01, 0.27)	0.05	0.087	0.02

β: Standardized regression coefficient; SE: Standard error; CI: 95% confidence interval; η^2^: Partial eta-squared (small = 0.01, medium = 0.06, large = 0.14). Hierarchical regression model: Adjusted for age, education, and years of service. Model assumptions: Normality (Shapiro–Wilk *p* > 0.05), no multicollinearity (VIF < 2.0). Compared with senior clinical nurses, *p <>0.05.

### 3.8 Multivariate regression analysis of mental health predictors

A hierarchical multiple regression model was constructed to examine the independent effects of occupational stress and coping strategies on mental health while controlling for demographic variables (Table 8). In Block 1, demographic variables explained 12% of the variance in SCL-90 scores (*F* = 8.21, *p* < 0.001). Younger age (β = 0.18, 95% CI [0.09, 0.27], *p* = 0.002) and lower education (β = 0.15, 95% CI [0.04, 0.26], *p* = 0.011) significantly predicted poorer mental health. Adding occupational stress in Block 2 increased the explained variance to 29% (ΔR^2^ = 0.17, *p* < 0.001), with occupational stress showing a strong positive association (β = 0.38, 95% CI [0.30, 0.46], *p* < 0.001). Finally, coping strategies in Block 3 accounted for an additional 9% of variance (ΔR^2^ = 0.09, *p* < 0.001), where maladaptive coping (β = 0.24, 95% CI [0.15, 0.33], *p* < 0.001) exacerbated mental health issues, while problem-solving coping (β = −0.19, 95% CI [−0.28, −0.10], *p* = 0.003) had a protective effect.

**Table 8:** Hierarchical regression analysis of factors influencing nurses’ mental health (SCL-90 total score).

Predictors	β (95% CI)	S.E	*p*	ΔR^2^	Effect size (η^2^)
Block 1: Demographics				0.12	0.15
Age	0.18 (0.09, 0.27)	0.04	0.002		
Education Level	0.15 (0.04, 0.26)	0.05	0.011		
Years of Service	−0.07 (−0.16, 0.02)	0.03	0.132		
Block 2: Occupational Stress				0.17	0.21
Work Stress Total	0.38 (0.30, 0.46)	0.04	<0.001		
Block 3: Coping Strategies				0.09	0.11
Problem-Solving	−0.19 (−0.28, −0.10)	0.04	0.003		
Maladaptive Coping	0.24 (0.15, 0.33)	0.04	<0.001		

β: Standardized regression coefficient; SE: Standard error; CI: Confidence interval; ΔR^2^: Incremental variance explained; η^2^: Partial eta-squared (small: 0.01, medium: 0.06, large: 0.14). Work Stress Total: Summed score from the Chinese Nurses’ Work Stress Source Survey Scale. Problem-Solving and Maladaptive Coping: Subscales of the Coping Strategies Scale. Hierarchical regression steps: Block 1 (demographics), Block 2 (occupational stress), Block 3 (coping strategies). Model assumptions met (normality: Shapiro–Wilk *p* > 0.05; homogeneity: Levene’s *p* > 0.10). ΔR^2^ values indicate significant incremental contributions (*p* < 0.001).

### 3.9 Mediation analysis of coping strategies in the occupational stress-mental health relationship

**Figure 1:**
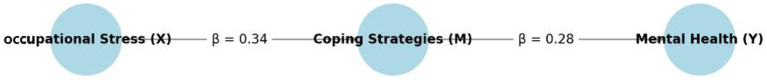
Mediation analysis model. This figure illustrates the relationships between occupational stress (X), coping strategies (M), and mental health (Y). The direct effect from occupational stress to coping strategies is β = 0.34, and the effect from coping strategies to mental health is β = 0.28. The indirect effect of occupational stress on mental health through coping strategies is also depicted with an indirect effect size of β = 0.18 (95% CI [0.11, 0.27]), accounting for 42% of the total effect.

The results revealed that occupational stress significantly predicted mental health issues (β = 0.41, *p* < 0.001). Coping strategies accounted for 42% of the total effect (indirect effect = 0.18, 95% CI [0.11, 0.27], *p* < 0.01). Specifically, occupational stress was found to significantly increase maladaptive coping (β = 0.34, *p* < 0.01), which, in turn, exacerbated mental health problems (β = 0.28, *p* < 0.01). The model demonstrated a good fit (χ^2^/df = 2.1, RMSEA = 0.06, CFI = 0.95), and a path diagram with standardized coefficients is presented in Figure 1.

## 4 Discussions

Our survey reveals that clinical nurses experience significant stress from multiple sources, including the inherent demands of the nursing profession, work-time allocation, workload, and patient care. In A-level hospitals, systemic factors such as low wage standards, limited career advancement, high task volumes, and restricted educational opportunities contribute substantially to nurse stress (3, 19).

Global studies further indicate that insufficient financial incentives and inadequate professional recognition exacerbate psychological distress among nurses (20). In Chinese hospitals, a pronounced shortage of nursing staff amplifies workloads and restricts opportunities for professional development, disadvantaging nurses relative to physicians (21). Additionally, inefficient time management and redundant non-medical tasks contribute to burnout and mental fatigue (22, 23). Recent research underscores the role of improved system management and process reengineering in alleviating these stressors (24).

The increased public awareness of healthcare quality has raised patient expectations, intensifying nurses’ decision-making burdens, especially in critical care environments where errors can have severe consequences (25). Current SCI evidence confirms that such high-pressure settings negatively affect both nurse well-being and patient care quality (20). Finally, the positive correlation between stress, age, and professional title suggests that cumulative workload and declining physical capacity, combined with limited growth opportunities, further drive stress among senior nurses (26). This finding is in line with global research calling for optimized work environments and fair promotion mechanisms to reduce stress and enhance professional satisfaction (12).

The concept of mental health refers to an individual’s psychological state, where their emotional and cognitive responses are balanced and adapted to environmental demands (9). In this study, clinical nurses exhibited significantly higher scores on the SCL-90 compared to Chinese norms, indicating poorer mental health among nurses. Most clinical nurses experience varying degrees of mental health challenges, which necessitates targeted interventions. This finding aligns with previous research by Zhang et, al (27), which highlighted that Chinese nurses face high professional pressures, poor mental health, and a growing need for psychological support.

Our findings validate the Transactional Model’s premise that coping strategies mediate stress-mental health relationships. The partial mediation suggests that interventions targeting adaptive coping could mitigate stress impacts. The study also found that surgical nurses scored significantly higher than both national standards and their peers in internal medicine on the SCL-90, indicating greater psychological distress. This is likely due to the nature of surgical nursing, where nurses deal with critically ill patients, high complication rates, and the rapid onset of conditions. The emotional strain of caring for patients in such dire circumstances, combined with frequent exposure to occupational hazards such as blood and bodily fluids, significantly increases the mental and physical health risks for surgical nurses (28). Similarly, nurses in intensive care units also reported elevated SCL-90 scores. ICU nurses face intensive workloads due to the high acuity of patients, the need for advanced technical skills, and the constant pressure to manage life-threatening situations. This contributes to both psychological stress and physical fatigue, which exacerbates mental health issues (29). Interestingly, the study found that senior nurses exhibited significantly higher scores across multiple dimensions of the SCL-90, including physicalization, obsessive-compulsive disorder, social sensitivity, depression, anxiety, phobia, paranoia, and general mental illness. Although senior nurses tend to have more experience and better communication skills, their inability to fully realize their career potential and the high-stress nature of their roles lead to dissatisfaction and burnout. These findings are consistent with previous studies that suggest senior nurses are often overburdened with critical care tasks and lack sufficient emotional support, leading to higher stress levels (30). Furthermore, the Pearson correlation analysis revealed that nursing profession stressors, time allocation, workload, and patient care are all positively correlated with higher SCL-90 scores, indicating a clear relationship between these factors and mental health issues. The multivariate logistic regression analysis also showed that work experience and professional titles significantly affect the mental health of nurses, particularly in high-stress areas such as surgery and intensive care. These factors should be taken into account when developing interventions to improve nurse well-being.

Occupational stress is defined as the physical and psychological strain experienced by employees due to the demands of their work and their perceived inability to meet those demands. For healthcare workers, particularly nurses, the psychological pressure is amplified due to the nature of their work, which is emotionally and physically demanding. Chronic stress can lead to serious emotional issues, negatively impacting both personal well-being and job performance (30).

Coping mechanisms play a crucial role in how nurses respond to stress. The study found significant differences in how various coping strategies affected stress levels. Nurses who employed positive psychological coping strategies were less affected by work pressure, whereas those who used negative coping mechanisms experienced higher stress levels (7). This suggests that when clinical nurses face high-intensity stress, adopting positive coping strategies—such as mindfulness, relaxation exercises, and seeking social support—can effectively mitigate negative emotions and improve overall mental health. Conversely, negative coping mechanisms, such as avoidance or emotional suppression, can lead to physical and mental exhaustion, exacerbating stress and negatively affecting both mental health and job performance (31).

Integrating modern psychological stress theory, we can view coping strategies as mediating factors between stress and mental health outcomes. Effective coping can enhance nurses’ problem-solving abilities, emotional regulation, and overall psychological adaptation, thereby improving their resilience in stressful work environments (32). Nurses should be encouraged to adopt positive coping strategies, engage in continuous learning and professional development, and maintain regular self-care practices to improve their mental well-being (33). These strategies are essential for promoting a healthier work environment and reducing burnout (34).

This study has several limitations that warrant consideration. First, despite efforts to stratify sampling by department and years of service, the reliance on convenience sampling within tertiary hospitals may introduce selection bias, as nurses who voluntarily participated might systematically differ from non-participants in stress perception or coping behaviors. Second, while hierarchical regression and mediation analyses were employed to address variable complexity, the cross-sectional design precludes causal inferences, and unmeasured confounders may influence outcomes. Third, the proposed stress reduction model, though validated in high-stress departments, requires cross-cultural adaptation and testing in diverse healthcare systems to ensure generalizability. Fourth, self-reported data may be susceptible to social desirability bias; future studies should integrate objective measures to triangulate findings. Lastly, while the sample size met statistical power requirements, the single-city focus limits extrapolation to rural or non-tertiary settings. To address these limitations, we recommend: (1) multi-center longitudinal studies tracking stress trajectories and coping efficacy over time; (2) mixed-methods designs combining quantitative surveys with qualitative interviews to contextualize coping mechanisms; (3) cross-cultural validation of the decompression model across regions with varying healthcare policies; and (4) experimental trials testing targeted interventions informed by this study’s findings. Such efforts will strengthen the evidence base for nurse well-being initiatives.

## 5 Conclusion

Positive coping strategies, such as mindfulness and social support, were associated with lower stress levels, while negative coping mechanisms exacerbated mental health challenges. However, the study’s limited sample size calls for further multi-center research to capture a more representative view. To improve nurse well-being and reduce burnout, it is essential to foster supportive work environments, promote positive coping strategies, and provide opportunities for professional development.

Our study found that clinical nurses in China experience considerable psychological distress as a result of their work, which significantly impacts their overall health and job satisfaction. The results also suggest that the use of positive psychological stress relief models can effectively alleviate stress, reduce physical and mental fatigue, and improve coping strategies. We recommend that hospitals prioritize implementing these models and invest in enhancing nurses’ psychological health to improve work efficiency and the quality of care provided. Future studies should explore the development of more targeted interventions for specific departments or stressors.

## Data Availability

The original contributions presented in the study are included in the article/supplementary material, further inquiries can be directed to the corresponding author.
